# A Mixed-Methods Evaluation to Inform the Hawaiʻi Suicide Prevention Strategic Plan

**DOI:** 10.3390/ijerph21050565

**Published:** 2024-04-29

**Authors:** Saikaew Dudla, Tarin T. Tanji, Jeanelle Sugimoto-Matsuda, Jane J. Chung-Do, Eric Agluba, Tricia Khun, Shivani Trivedi, Deborah Goebert

**Affiliations:** 1 Office of Public Health Studies, Thompson School of Social Work & Public Health, University of Hawaiʻi at Mānoa, Honolulu, HI 96822, USA; tarin_t_tanji@rush.edu (T.T.T.); jsugimot@hawaii.edu (J.S.-M.); chungjae@hawaii.edu (J.J.C.-D.); eric.agluba@wsu.edu (E.A.); tkhun@hawaii.edu (T.K.); shivanit@hawaii.edu (S.T.); goebertd@dop.hawaii.edu (D.G.); 2 Department of Psychiatry, John A. Burns School of Medicine, University of Hawaiʻi at Mānoa, Honolulu, HI 96822, USA

**Keywords:** suicide, suicide prevention, program evaluation, community action, suicide policy, suicide strategy, organizational objectives, Hawaiʻi, mixed methods, suicide prevention training

## Abstract

The Prevent Suicide Hawaiʻi Taskforce is a state, public, and private partnership of individuals, organizations, and community groups that leads statewide suicide prevention efforts in Hawaiʻi. The purpose of this evaluation was to identify the progress and barriers of the Taskforce to inform the upcoming 2025 Hawaiʻi Suicide Prevention Strategic Plan in the following areas: Hope, Help, Heal, Research and Evaluation, and Policy and Advocacy. Utilizing a sequential exploratory mixed-methods approach, 18 key informants were interviewed, followed by a 13-question survey sent to the Taskforce member listserv. Results were analyzed using qualitative coding techniques and descriptive statistics. Interview findings contained six themes: importance of community relationships, interconnection of suicide prevention efforts, progress in diversifying training, organizational challenges, adaptations to the COVID-19 pandemic, and funding challenges. Of the 34 survey respondents, most were involved in the area of Hope (91%). The respondents reported the area with most progress was Hope (87%), and the most important area to address was Help (41%). The majority (82%) of the respondents characterized the level of Taskforce communication as Excellent or Good. Interview and survey data corroborated each other and revealed new insights about the successes and barriers of the Taskforce and their progress in implementing the Strategic Plan. Recommendations included advocating for long-term funding for suicide prevention and building community relationships.

## 1. Introduction

### 1.1. Suicide in the United States

Suicide is a serious public health issue. In 2020, there were 45,979 deaths by suicide, 1.2 million suicide attempts, and an age-adjusted suicide rate of 13.48 per 100,000 individuals, making it the 12th leading cause of death in the United States [1]. The risk of suicide can be influenced by a combination of factors, ranging from individual and relationship factors, such as a history of depression and bullying, to community and societal factors, such as historical trauma, discrimination, and stigma associated with mental illness [2].

### 1.2. Suicide in Hawaiʻi

Studies have shown that in Hawaiʻi, a high level of family cohesion, positive friendships, and belonging to a community that enhances connection and care are protective factors against suicide [3,4]. Nevertheless, suicide remains a leading cause of death in Hawaiʻi, with an age-adjusted rate of 12.9 deaths per 100,000 in 2021 [5,6]. Similar to other health issues, there are disparities in suicide trends among certain subgroups in Hawaiʻi. Health disparities, in general, are rooted in structural inequities that negatively impact specific groups of people [7]; this is especially salient to recognize in Indigenous communities who experience collective trauma, discrimination, and land loss from colonization [8]. Between 2017 and 2021, Native Hawaiians and Other Pacific Islanders had a higher age-adjusted suicide death rate (17.1 and 20.6) compared to the overall population in Hawaiʻi (13.1) [9]. Similar to national patterns, individuals living in rural counties in the state of Hawaiʻi also have a higher risk of suicide compared to those that live in urban areas, with the highest age-adjusted rates in Hawaiʻi County (20.9), followed by Maui County (17.6), Kauaʻi County (13.0), and Honolulu County (11.1), between 2019 and 2021 [10]. Overall, youth in Hawaiʻi are also at a higher risk, with suicide being the second leading cause of death among youth of ages 15–24 and 25–34 years and the fourth leading cause among ages 1–14, 35–11, and 45–54 years [11].

### 1.3. Prevent Suicide Hawaiʻi Taskforce

In 2001, the Prevent Suicide Hawaiʻi Taskforce (Taskforce) was established as a partnership of individuals, community groups, and organizations who work together to bring attention to suicide prevention in Hawaiʻi [12]. The Taskforce now includes a diverse group of members from over 100 different public and private organizations across Hawaiʻi [13]. The Taskforce encompasses the social and cultural contexts in Hawaiʻi to develop policy, legislation, and strategic planning for suicide prevention. Within the statewide Taskforce, there are four county-specific Taskforces on Oʻahu, Kauaʻi, Hawaiʻi Island, and Maui County, which includes the islands of Maui, Molokaʻi, and Lānaʻi.

### 1.4. The State’s Strategic Plan

In 2017, the Taskforce developed the State of Hawaiʻi’s Strategic Plan on Suicide Prevention (referred to as Strategic Plan), intending to reduce suicide in Hawaiʻi by at least 25% by 2025 [13]. This document was submitted to the Hawaiʻi State Legislature on December 28, 2017. The five areas of the Strategic Plan are Hope, Help, Heal, Research and Evaluation (Research), and Policy and Advocacy (Policy) (see Table 1). Hope focuses on prevention by increasing community awareness around suicide prevention and improving statewide capacity for training. For example, Hawaiʻi’s Caring Communities Initiative for Youth Suicide Prevention works to raise suicide awareness and promote protective factors through workshops on hula, lei-making, and other culturally relevant activities [3]. The second strategy, Help, ensures that suicide prevention is a core component of Hawaiʻi’s overall system of care. The University of Hawaiʻi, John A. Burns School of Medicine, Department of Psychiatry (JABSOM-DOP) is a leader in providing suicide prevention training, such as the Connect Suicide Prevention Program and SafeTalk to healthcare providers and the community [3]. JABSOM-DOP has also adopted the Zero Suicide Conceptual Approach and Practice framework to its healthcare system to improve outcomes for those at risk for suicide. Services provided include suicide screening, assessment, and treatment [3]. The third strategy, Heal, aims to increase community capacity to effectively and efficiently respond to individuals and communities affected by suicide. Research is the fourth strategy that informs suicide prevention programs, interventions, policies, and overall Statewide direction. The last strategy, Policy, ensures that policies and protocols set the proper foundation for suicide prevention initiatives.

Current trends show suicide rates in Hawaiʻi have varied each year, though not significantly, since the inception of the Strategic Plan, from 14.5 deaths per 100,000 (95% CI 12.6, 16.4) in 2017 to 11.3 (95% CI 9.6, 13.0) in 2018, 15.0 (95% CI 12.9–17.0) in 2019, 11.8 (95% CI 10.0, 13.6) in 2020, and 12.9 (95% CI 11.0, 14.8) in 2021 [5,6]. Additionally, although the Taskforce has been heavily involved and active in suicide prevention efforts, there has yet to be an evaluation of its progress and priorities as they relate to the Strategic Plan. Therefore, an evaluation was conducted to ensure that the Taskforce continues to make maximum impact on suicide prevention in Hawaiʻi, according to the members’ input. This evaluation prioritized data collection of member experiences and knowledge regarding the Taskforce because engaging stakeholders throughout evaluation processes increases the relevancy of findings and ensures that recommendations are more likely to be adopted by the organization [14]. The results from this evaluation will inform the second iteration of the Strategic Plan in 2025.

### 1.5. Theoretical Framework

Since the Taskforce operates around the five strategies of the Strategic Plan, the evaluation utilized them as the overarching framework. An exploratory sequential, mixed-methods approach through interviews with select Taskforce stakeholders, followed by a survey with the larger Taskforce was deemed appropriate for this evaluation. The qualitative approach for the first phase of the evaluation (interviews) identified salient themes with regard to the five Strategic Plan areas. These themes were then incorporated into the survey to gauge generalizability among the larger Taskforce members [15]. The qualitative and quantitative data results were then integrated to provide a richer understanding of the results [15].

### 1.6. Project Purpose and Plan

This evaluation aimed to inform the Strategic Plan’s upcoming review by conducting individual and small group interviews with key Taskforce members and a survey with the entire Taskforce. The evaluation aimed to answer three evaluation questions:

- What successes and barriers has the Taskforce faced?
- What is the level of involvement, progress, and future priorities for the Taskforce in each area of the Strategic Plan (Hope, Help, Heal, Research, and Policy)?
- What is the level of collaboration and communication in the Taskforce?

## 2. Materials and Methods

### 2.1. Study Design

**Figure 1:**
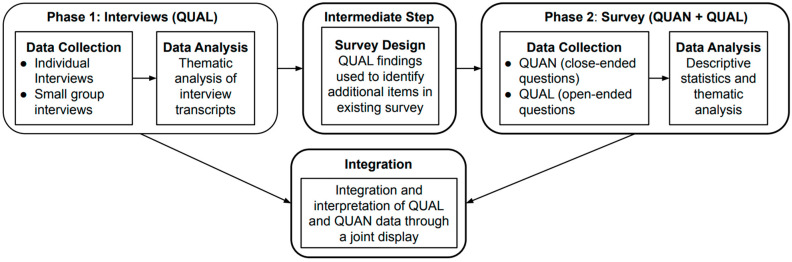
Study design.

This evaluation employed a sequential exploratory mixed-methods approach [15]. A mixed-methods approach involves the separate collection and analysis of qualitative and quantitative data [15]. This is followed by the integration of the two datasets to gain a more comprehensive understanding of the problem, typically represented graphically by a joint display [15]. Specifically, a sequential exploratory design begins with the collection of qualitative data (Phase 1) to explore a phenomenon when the problems are not well understood; an instrument (a survey) is then developed based on those findings (Intermediate Step) and then implemented on a larger sample (Phase 2) [15]. This evaluation contained three phases: Phase 1 involved individual and small group interviews to identify salient themes that informed the development of a survey (Intermediate Step) implemented among the entire Taskforce in Phase 2 (see Figure 1).

### 2.2. Participants

#### 2.2.1. Phase 1 (Individual and Small Group Interviews)

Taskforce members and/or stakeholders involved in suicide prevention in Hawaiʻi were eligible for this phase of the evaluation. A purposive sampling technique was used, where Taskforce steering committee members recommended potential participants to be interviewed individually or in a small group, depending on the nature of their role. Steering committee members hold key positions on the County Taskforces, within State agencies, or are key partners in implementing the Strategic Plan. They identified 19 individuals who were considered knowledgeable about suicide prevention activities in the State or a County of Hawaiʻi. Participants who are involved in the same initiatives/workgroups were interviewed in small groups. This helped ensure insights were grounded in a common context and reduced the number of required interviews and redundancy between interviews.

#### 2.2.2. Phase 2 (Survey)

All Taskforce members and/or stakeholders involved in suicide prevention in Hawaiʻi were eligible for this phase. Members have signed up to receive monthly correspondence about suicide prevention activities. Some members also participate in training and networking opportunities offered every other month at Taskforce meetings.

### 2.3. Measures

#### 2.3.1. Phase 1 (Individual and Small Group Interviews)

The evaluation team collaboratively wrote a semi-structured interview script that aimed to elicit information of the current state and direction of the suicide prevention movement in Hawaiʻi, the progress being made with respect to each area of the Strategic Plan, and what contributes to or prevents progress. The interview script contained opening, transition, key, and summary questions, for a total of 17 questions [16].

#### 2.3.2. Intermediate Step (Survey Development)

The survey consisted of 13 Likert-scale, six multiple-choice, and four open-ended questions (Supplementary Figure S1). The main findings from Phase 1 (interviews) prompted further investigation into Taskforce members’ perceptions about the progress being made in the various areas of the Strategic Plan, which areas deserve priority, and the level of involvement, collaboration, and communication in the Taskforce. The six questions regarding the assessment of progress, priority, and involvement were drawn from the Taskforce’s 2017 Strategic Planning Member Survey, an instrument that has not been psychometrically tested, yet was developed collaboratively by leaders of the Taskforce following the Strategic Plan to ensure content validity. The two questions about communication and collaboration were taken from the 2022 State and Territorial Suicide Prevention Evaluation Survey [17] and the Levels of Collaboration Scale [18]; the former is a nationally utilized survey conducted annually since 2021 [17], and the latter is a collaboration scale that has been tested for reliability and validity [18]. The remaining five questions asked for demographic information and an open-ended inquiry for final comments.

### 2.4. Procedures and Analysis

#### 2.4.1. Phase 1 (Individual and Small Group Interviews)

Each potential participant received an email that contained the purpose and process of the evaluation, the consent process, and potential meeting dates. The evaluation team scheduled 30–90 min individual and small group interviews, then emailed a Summary of the Strategic Plan (Supplementary Table S1) and a Zoom meeting link.

Each interview began with personal introductions, the cultural practice of “talking story”, and an icebreaker. The interviewers then reiterated the purpose and process of the study. During the informed consent process, individuals were asked for verbal consent for permission to (1) audio record the interview or small group and (2) attach their name to their responses in the results. If the individual did not consent to either, the research team took notes and omitted any identifying information from their data. Following the consent process, the team conducted the individual or small group interviews with the interview script. Each interview was 60 min on average. Each participant received a note and gift card as a thank-you for their involvement.

Audio recordings were transcribed verbatim by the evaluation team. After the transcriptions or notes were sent to participants for approval, the recordings were deleted. Interviews were individually coded using an inductive coding technique for ideas related to the Taskforce’s progress and barriers, and each code was compiled into a spreadsheet. Reliability was ensured through investigator triangulation of multiple collaborative discussions, where each code was reviewed and grouped into themes. Overall, six themes were identified. Validity of the themes was ensured when the investigators sought feedback from the Taskforce in their dissemination efforts. These findings were utilized to make recommendations for the Taskforce.

#### 2.4.2. Phase 2 (Survey)

Emails were sent to Taskforce leaders who served on the steering committee on 10 October 2022. The recipients were asked to forward this email with the Google Forms survey link to their respective members on the Taskforce listserv, which included 239 individuals. The first page of the Google Forms survey contained the consent form, and participants provided their consent by proceeding to the next page. Three reminder emails were sent on 24 October, 31 October, and 7 November 2022. The survey was closed on 15 February 2023.

The survey results were compiled into a spreadsheet. Descriptive statistics such as frequency and percentages were used to summarize Likert-scale responses. Open-ended questions were coded individually and then compiled into themes as a group.

#### 2.4.3. Integration of Phase 1 and 2 Databases

Phase 1 (interviews) and Phase 2 (survey) databases were integrated systematically to confirm and elaborate on findings; this process was visualized in a joint display table. For each evaluation question, qualitative results from interviews were listed in the first column of the table, and matching quantitative and qualitative results from the survey were listed adjacent in the third and fourth columns. The evaluation team independently determined whether the data in these three columns confirmed, elaborated on, or refuted each other and collaboratively produced the fourth column, the final interpretation of the combined data.

## 3. Results

### 3.1. Phase 1 (Individual and Small Group Interviews)

A total of 18 individuals participated in five individual and five small group (three groups of two and three groups of three) interviews from 21 October 2021 to 19 November 2021. Collectively, participants spent an average of 11 years involved with the Taskforce. We identified six themes from the interviews (Table 2).

#### 3.1.1. Theme 1: Suicide Prevention Efforts Are Centered on Building and Sustaining Relationships and Partnerships with the Community to Achieve Goals

Suicide prevention is an ongoing, community-centered process that is focused on building and sustaining relationships. Most interviews mentioned that the Taskforce valued diversity and acknowledged that every member has their own strength and specialty that contribute to the overarching mission of suicide prevention. One participant stated the following:

*“I think we all come out as a collective impact. We do not do things individually. It’s strict collaboration… and that’s what the Taskforce is all about... We work collaboratively, we work as partners collectively, and we support each other.”*

Furthermore, most interviews mentioned that building and sustaining relationships is fundamental to achieving organizational objectives and overcoming challenges, such as sharing resources to counter the lack of funding. One participant stated,

*“In general, successful policy and advocacy can be achieved by building bridges and fostering kind and helpful relationships. It is easy to react to an event, but it’s much harder to grow roots. The role of us is to foster, grow, and identify new partnerships.”*

#### 3.1.2. Theme 2: The Taskforce Views Suicide Prevention in a Holistic, Interconnected, and Cyclical Way

Many interviews mentioned that approaches to suicide prevention should take a holistic perspective. From the health service context, participants stated the importance of continuity of care and the inextricable link between physical and mental health. One participant stated,

*“...make sure that those providers are talking to each other, they’re not working in silos. And so if the primary care doctor knows that they’re seeing someone for mental health and the mental health person can call to make sure that, you know, all these different things they’re seeing are also being addressed so they can just have those beautiful discussions.”*

Participants also spoke about how each of the Strategic Plan’s five strategies should not be viewed or tackled in isolation because they feed into and impact one another. One participant stated,

*“That’s what strategy three (Heal) was meant to be, that after a suicide occurs in postvention, to really build up our ability to not just have these steps of progress, but to respond because we say good postvention (Heal) is actually good prevention (Hope). Because when you do a good response, then it actually helps to reduce risk for the people in that family or in that community.”*

#### 3.1.3. Theme 3: There Has Been Much Progress in the Number and Types of Suicide Prevention Training for Diverse Populations

There was high consensus among the participants that suicide prevention training in Hawaiʻi has progressed in both quantity and types of training offered. These include SafeTALK, ASIST, and Youth Mental Health First Aid training. These training courses educate about suicidal risk and protective factors, teach resiliency and coping skills, and connect the community to resources.

The Taskforce has partnered with numerous organizations such as the Hawaiʻi Department of Education and police departments to train their employees, including teachers, counselors, administrators, and police officers. In the wider community, the participants spoke about how the Taskforce strives to ensure that all training and outreach programs are culturally relevant to the diverse population of Hawaiʻi. One participant stated,

*“Really approaching cultural diversity is such a huge thing. I mean, we’re in Hawaiʻi, we’re such a diverse cultural state. We should all be respectful of everyone’s culture.”*

Despite the progress, the participants reported that there is still room for improvement to ensure that training efforts strike a balance between being culturally aligned and evidence-based:

*“We are constantly getting feedback from our evidence-based trainings, that they are not aligned with the culture here.”*

And,

*“How do we approach, you know, what is culturally relevant for them? You know, everything from eye contact or lack of eye contact, body, language, face-to-face side, you know, all those kinds of things we need to take into consideration.”*

#### 3.1.4. Theme 4: The Taskforce Faces Challenges with Organization in Statewide Suicide Prevention Effort

Though Taskforce diversity is a source of strength in achieving collective impact, many interviews also noted the lack of a unifying framework for maintaining organization of the Taskforce’s activities, including events to raise awareness about suicide prevention and safe messaging, suicide prevention trainings, and maintaining the Hawaiʻi Coordinated Access Resource Entry System (Hawaiʻi CARES).

One participant stated,

*“I love the group that’s attracted to this. But we also could get a little more organized if we’re going to join together and really take it to the next level because so much has already happened. So we’ve got a foundation, now we’ve got to really get it together for what’s next.”*

Multiple interviews mentioned that the Taskforce needs a more organized postvention system to ensure that coordinated services are continuously available to the community. One participant stated,

*“It can’t just be that handful of people…that is able to provide that support and provide that [postvention] response. So then from a system stand point, how do we build that better? So that we have providers in the state that are capable of doing such a thing, but how do we connect that all?”*

#### 3.1.5. Theme 5: COVID-19 Has Presented Challenges to Hawaiʻi’s Communities and the Taskforce—However, the Taskforce Has Responded with Strength, Creativity, and Adaptability to Continue Its Suicide Prevention Efforts

The COVID-19 pandemic presented challenges to the suicide prevention movement and Taskforce. The pandemic decreased the social connectedness of the Taskforce and community members, as they could no longer hold training, meetings, and suicide survivor events in person. Another challenge for the community was the increase in mental health issues, including depression and anxiety.

One participant stated,

*“We’re at a higher risk this year than we have been in the past. And I really have to attribute that to the isolation and COVID. I think that COVID has really affected a lot of people. We have more suicides right now to date than we did last year for the whole year. We still have more than a month-and-a-half to go. And it’s such an at-risk time, even with the ebbs and the flows of what we’re seeing in our community.”*

However, COVID-19 also highlighted the community’s strengths, creativity, and ability to adapt to uncertain situations presented by the pandemic. One participant stated,

*“But yet we continued those conversations about what everyone was doing and adapting, which was very helpful … so at that point, I think it was a lot of brainstorming and supporting one another. And we’re very thankful to have had that … and even when the resources seemed to get smaller and tighter, we still would say, ‘How can we partner in really creative ways? Who have I not worked with? How can we use what we have to make the most out of it?’ And out of this, again, an unexpected silver lining was really creative partnerships.”*

#### 3.1.6. Theme 6: The Taskforce Faces Challenges with Long-Term and Sustainable Funding

The Taskforce successfully secured funding from the Substance Abuse and Mental Health Services Administration. However, most of the interviews mentioned the need for more long-term funding to maintain efforts for training, employee and volunteer compensation, research, evaluation, and more. One participant stated,

*“The Taskforce persists through all these years, but let’s face it. How much can you do with no funding versus big funding? It has to be something sustainable, and we can’t just continue relying on these rolls the dice, cross your fingers, kind of federal grant opportunities. The State really has to put that investment in too so that we’re not constantly in this kind of up-and-down thing with the resources.”*

### 3.2. Phase 2 (Survey)

The survey was sent to all members of the Taskforce listserv, which included 239 individuals. Out of these individuals, 58 members were considered active and attended multiple meetings per year. A total of 34 participants responded to the survey, which is a 59% response rate (see Table 3). Most survey respondents (62%) were 45 to 64 years of age and identified as female (59%). The geographic representation was as follows: Honolulu County (62%), Kauaʻi County (15%), Hawaiʻi County (12%), and Maui County (9%). The survey respondents were a diverse group of community members who represented various organizations or groups.

#### 3.2.1. Involvement, Progress, and Importance

Survey respondents reported on their level of involvement with each strategy in the Strategic Plan (Table 4). The strategies of Hope and Help had the highest level of involvement, with the majority of participants reporting that they were “Already very involved” (65% and 47%, respectively). Heal and Policy had more auxiliary engagement from participants, with the majority reporting that they “Assist/support others” (53% and 44%, respectively). Research had the lowest levels of involvement, with an equal number of participants who were “Not involved but would like to be” (26%) and “Not involved, and not my area of focus” (26%).

Survey respondents reported on the progress seen in each strategy (Table 4). Hope had the highest percentage of participants reporting some or a lot of progress (88%), followed by Help (81%), Policy (75%), Research (60%), and Heal (57%).

Survey respondents ranked the strategies in order of importance (Table 4). Help was ranked as first priority (41%), followed by Hope (24%), Heal (18%), Policy (15%), and Research (12%). Table 5 displays the summary of involvement, progress, and importance with Hope and Help being the most mentioned areas of the Strategic Plan. For the strategy that respondents ranked as the most important, they were asked to suggest a specific action step to make progress (Table 6). The most mentioned suggestion was to increase education through providing more classes, training, and resources.

#### 3.2.2. Successes and Barriers

Survey respondents elaborated on their greatest success with the strategies they were involved in. Of the 29 comments, 38% related to success in collaboration and community-building, 38% to success in training, 34% to increase suicide prevention awareness and outreach, 10% to obtaining funding, and 7% to increasing research and evaluation. One respondent said,

*“[My greatest success is] increasing my knowledge related to suicide awareness, as well as being able to share with others in our community of first responders along with various other stakeholders.”*

Twenty-nine respondents commented on their greatest barrier in the strategies they were involved in. Themes that emerged included lack of funding, time, and resources (45%), challenges with outreach (14%), policy and organizational challenges (10%), lack of connection with communities (10%), personal difficulties (7%), difficulty working with providers (3%), and difficulty working with survivors or individuals in a suicidal crisis (3%). As one respondent commented,

*“[The greatest barriers are] time, money, resources, and the need to duplicate myself….”*

#### 3.2.3. Communication and Collaboration

Approximately 44% of respondents characterized the overall level of communication within the Taskforce as Excellent (Table 7). In relation to collaboration (Table 8), 29% selected Coalition, and 24% selected Coordination. The highest level, Collaboration, was selected by 12% of respondents.

#### 3.2.4. Open-Ended Survey Responses

Seventeen respondents provided final comments. Themes that emerged included words of appreciation for the Taskforce (47%), words of appreciation for the evaluation (41%), and the need for more funding (18%). One respondent stated,

*“I love this Taskforce and all of its accomplishments made throughout the years. I believe it has been helpful in getting awareness to our community and empowering many to give hope and help.”*

### 3.3. Integration of Results

Integration of the Phase 1 (interviews) and Phase 2 (survey) databases revealed a confirmation of findings and new insights into the Taskforce and the Strategic Plan (Table 9). For the first evaluation question regarding successes and barriers, survey results confirmed the interview finding that there is much progress in the number of suicide prevention trainings available for diverse populations. However, the survey findings also revealed there is still room for growth for creating stronger connections with communities in Hawaiʻi. For barriers, there was a general consensus that increasing funding remains a top priority. For the second evaluation question regarding the five Strategic Plan areas, survey results confirmed interview findings that there was high involvement, progress, and prioritization of Hope and Help, and lower levels for Policy. While interview participants recognized the importance of Research, this area ranked low in involvement, progress, and priority in the survey. For Heal, while interviewees mentioned many events and resources for survivors, this area was ranked last in priority in the survey. For the third evaluation question, survey results confirmed interview findings that communication and collaboration is important but revealed that there is room for improvement in collaboration.

## 4. Discussion

This evaluation aimed to learn the successes and barriers that Taskforce members experienced in their suicide prevention work in relation to the Strategic Plan. Through the interviews, we identified six themes. Survey results showed a high level of communication and collaboration within the Taskforce. The most progress was seen in Hope (89%) and Help (82%), and the least progress was in Research (61%) and Heal (58%). The Taskforce’s greatest successes revolved around collaboration and community-building, and the greatest barriers were related to a lack of funding, time, and resources.

Our finding that partnerships and relationships are the Taskforce’s greatest strengths aligns with previous research that suicide prevention efforts in Hawaiʻi require a strong foundation of trusting relationships [19,20]. In a study that interviewed community leaders and trainers, prioritizing relationships was identified as a key cultural need for the successful implementation of a suicide prevention program in Hawaiʻi communities [19]. The emphasis on fostering relationships likely contributes to the high level of progress seen in Hope and Help, which focuses on outreach and training, two areas that require strong interpersonal and community trust and understanding. The importance of relationship building is also highlighted in Goebert et al.’s 2018 study, where the authors emphasize four Native Hawaiian values at the core of suicide prevention work [20]. Cultivating the Hawaiian value of pilina, which highlights the importance of building connections and relationships, can enhance community resilience and help individuals navigate difficult times [19]. The community’s perseverance was demonstrated when the COVID-19 pandemic introduced many challenges to suicide prevention efforts.

Our finding on the Taskforce’s challenges related to lack of funding, resources, staff, and time is an issue faced by global and local suicide prevention efforts. Although suicide is a national leading cause of death, suicide prevention efforts are severely underfunded [21]. A scoping review of 64 studies on the facilitators and barriers of suicide prevention interventions found that a common barrier is a lack of time to engage in efforts because staff and volunteers have very demanding schedules [22]. When coupled with a lack of funding to compensate for these efforts, it creates a burden on suicide prevention advocates. Similarly, a local youth suicide prevention effort stated that long-term sustainability was an issue because their funding was awarded for only one year [23].

### 4.1. Recommendations

Based on the identified themes, the evaluation team made four recommendations to the Taskforce. The first is to continue to advocate for local governments to invest in long-term, sustainable funding for suicide prevention so the Taskforce can provide more community training, mental health resources, and compensation for members and volunteers. Second, the Taskforce can continue to build relationships within the Taskforce and community through effective collaboration and communication. This includes strengthening relationships with community leaders to advocate for legislation and funding for suicide prevention. Third, the Taskforce can gauge the level of interest in strategies that many Taskforce members are not currently involved in (Research and Policy) by conducting another survey with a larger sample. Lastly, the Taskforce can develop standard evaluations and research to track the progress of suicide prevention in Hawaiʻi.

### 4.2. Dissemination

Interview and survey findings were presented at two monthly Taskforce meetings on 16 December 2021 and 20 April 2023. The findings were well-received by Taskforce members, and the Taskforce restructured meetings and prioritized policy to more closely align with the Strategic Plan.

### 4.3. Strengths and Limitations

This evaluation used a mixed-methods approach, which provided rich, contextual information from a small number of key informants, along with feedback from the larger Taskforce. Additionally, the survey was adapted from the Taskforce’s 2017 Strategic Planning Member Survey, which can continue to be used in subsequent evaluations to allow for analysis over many years.

This evaluation had several limitations. First, it primarily relied on the experiences and knowledge of Taskforce members and does not include the wider public who are impacted by suicide. While this can be a limitation, member opinion is highly valuable in informing the future direction of the Taskforce; it provides direction for further research incorporating the perspectives of other members of the community, which is expanded on in the following section. In addition, the survey sample size (*n* = 34) was small compared to the total number of people on the Taskforce listservs. However, this was unlikely to affect evaluation findings, given that the response rate was acceptable at 59%, when comparing it to the 58 active Taskforce members, and that a mixed-methods approach was utilized to triangulate the survey and interview findings. Question 13 of the survey had a high non-response rate (53%), likely because it was the last question that asked for “any final comments”. Otherwise, missing data were not an issue for the rest of the survey questions, with high response rates between 82 and 100%. Questions with rates on the lower end were all open-ended. 

## 5. Conclusions

This evaluation highlights the successes and challenges of suicide prevention efforts in Hawaiʻi and provides recommendations for the second iteration of the Strategic Plan in 2025. Future research considerations include developing formal and ongoing implementation and outcome evaluations with wider samples and objective measures to better understand the impact of Taskforce initiatives in Hawaiʻi.

## Figures and Tables

**Table 1:** Strategic plan areas.

Strategy	Goal(s)
Strategy 1: Hope	1a. Increase community awareness and communication around suicide prevention as a public health problem that is preventable. 1b. Increase statewide capacity for training across multiple levels and disciplines, including a focus on cultural humility with diverse populations.
Strategy 2: Help	2. Promote suicide prevention as a core component of Hawaiʻi’s overall system of care.
Strategy 3: Heal	3a. Increase Hope, Help, Healing, and Wellbeing among those personally touched by suicide and among those with lived experience. 3b. Increase State and Community capacity to effectively and efficiently respond to individuals and communities affected by suicide and those with mental health challenges.
Strategy 4: Research and Evaluation	4. Conduct and support high- quality research and evaluation to inform suicide prevention programs, interventions, policies, and overall Statewide direction.
Strategy 5: Policy and Advocacy	5. Ensure policies and protocols set the proper foundation for suicide prevention initiatives.

**Table 2:** Interview themes.

Interview Themes
Suicide prevention efforts are centered on building and sustaining relationships and partnerships with the community to achieve goals. The Taskforce views suicide prevention in a holistic, interconnected, and cyclical way. There has been much progress in the number and types of suicide prevention trainings for diverse populations. The Taskforce faces challenges with organization in statewide suicide prevention efforts. COVID-19 has presented challenges to Hawaiʻi’s communities and the Taskforce. However, the Taskforce has responded with strength, creativity, and adaptability to continue its suicide prevention efforts. The Taskforce faces challenges with long-term and sustainable funding.

**Table 3:** Prevent Suicide Hawaiʻi Taskforce evaluation survey participant demographics.

Variable	N (%)
Age Group	
44 years and under	9 (27)
45–64	21 (62)
65 and over	3 (9)
No response	1 (3)
Gender	
Female	20 (59)
Male	10 (29)
Other ^1^	4 (12)
County	
Honolulu	21 (62)
Kauaʻi	5 (15)
Hawaiʻi	4 (12)
Maui	3 (9)
No response	1 (3)
Organization/Group ^2^	
Individual from community	26 (77)
Community/nonprofit or faith-based organization	15 (44)
State Agency	9 (27)
Military	9 (27)
Education	7 (21)
Healthcare organization	6 (18)
Business	4 (12)

^1^ Transgender, non-binary/non-conforming, other, prefer not to respond/no response. ^2^ Participants were asked to select all that apply.

**Table 4:** Level of involvement, progress, and importance of each strategy.

Level/Rank			Strategy (%)		
	Hope	Help	Heal	Research	Policy
Involvement					
Already very involved	65	47	21	24	24
Assist/support others	26	38	53	24	44
Not involved, but would like to be	9	9	21	26	18
Not involved, and not my area of focus	0	6	6	26	15
Progress					
A lot of progress	52	39	15	21	42
Some progress	36	42	42	39	33
A little bit of progress	12	18	33	36	21
No progress at all	0	0	9	3	3
Importance					
1st	24	41	18	12	15
2nd	35	21	6	15	26
3rd	12	21	38	21	12
4th	18	12	18	18	29
5th	12	6	21	35	18

**Table 5:** Summary of involvement, progress, and importance.

	Involvement ^1^ (%)	Progress ^2^ (%)	Importance ^3^ (%)
1st	Hope (91)	Hope (88)	Help (41)
2nd	Help (85)	Help (82)	Hope (24)
3rd	Heal (74)	Policy (76)	Heal (18)
4th	Policy (68)	Research (61)	Policy (15)
5th	Research (47)	Heal (58)	Research (12)

^1^ % = Already very involved and Assist/support others. ^2^ % = A lot of progress and Some progress. ^3^ % = 1st.

**Table 6:** Specific action steps to make progress.

Specific Action Step	N (%)
Increase education through providing more classes, trainings, resources	13 (43)
Continue promoting positive messages of resilience/awareness	4 (13)
Foster ongoing partnerships within the Taskforce and community	4 (13)
Increase the number of mental healthcare providers and services	4 (13)
More research and evaluation	4 (13)
Increase advocacy efforts to change policy and gain more funding	4 (13)

**Table 7:** Levels of communication within the Taskforce.

Communication	N (%)
Excellent	15 (44)
Good	13 (38)
Fair	3 (9)
Poor	1 (3)
Extremely Poor	0 (0)
No answer	2 (6)

**Table 8:** Levels of collaboration within the Taskforce.

Collaboration	Description	N (%)
Collaboration	Members belong to one system; consensus is reached on all decisions	4 (12)
Coalition	Share ideas; all members have a vote in decision making	10 (29)
Coordination	Share information and resources; some shared decision making	8 (24)
Cooperation	Provide information to each other; decisions are made independently	3 (9)
Networking	Aware of each other’s activities; decisions are made independently	4 (12)
None	No awareness or interaction	2 (6)
No answer	Respondent did not answer	3 (9)

**Table 9:** Joint display.

Phase 1 (Interviews)		Phase 2 (Survey)			→	Interpretation of Combined Data
Qualitative Results	+	Quantitative Results	+	Qualitative Results		
Evaluation Question 1: What successes and barriers has the Taskforce faced?						
There has been much progress in the number and types of suicide prevention trainings for diverse populations.		Involvement: Hope (91%) and Help (85%) Progress: Hope (88%) and Help (81%)		Comments on greatest success: 38% related to collaboration and community-building Comments on greatest barriers: 14% outreach, 10% lack of connection with communities		There has been much progress in providing different kinds of suicide prevention training, but there is still room for growth by connecting with more communities in Hawaiʻi.
The Taskforce faces challenges with organization in statewide suicide prevention efforts.		Level of communication: good to excellent (82%). Level of collaboration: High (41%), moderate (24%), low (21%), and none (6%)		No comments related to this theme.		Interviews identified potential issues with the Taskforce’s organization. However, survey results indicated the majority of participants thought the Taskforce had good-to-excellent communication and moderate-to-high levels of collaboration.
COVID-19 presented challenges to Hawaiʻi’s communities and the Taskforce. However, the Taskforce responded with strength, creativity, and adaptability to continue its efforts.		Not measured.		No comments related to this theme.		COVID-19 presented challenges, but the Taskforce responded with creativity and adaptability.
The Taskforce faces challenges with long-term and sustainable funding.		Policy: Involvement (68%), Progress (76%), Importance (15%)		Comments on greatest barrier: 45% cited lack of funding, time, and resources Specific Action Steps: 13% cited increased advocacy efforts to change policy and gain more funding		Lack of funding is a significant issue raised in both the interviews and the survey.
Evaluation Question 2: What is the level of involvement, progress, and future priorities for the Taskforce in each area of the Strategic Plan						
Hope: The Taskforce is involved in multiple suicide prevention events to raise awareness. Social media addresses stigma and raises awareness through safe messaging.		Ranking of Hope: Involvement: 1st Progress: 1st Priority: 2nd		Specific Action Steps: 13% cited continue promoting positive messages of resilience and awareness. Greatest successes: 38% in training, 34% increase in suicide prevention awareness and outreach		There is a lot of engagement and prioritization for Hope.
Help: The Hawaiʻi Coordinated Access Resource Entry System has improved over the years, but can be made more accessible. There is a need for more services and community health providers in Hawaiʻi’s healthcare system.		Ranking of Help: Involvement: 2nd Progress: 2nd Priority: 1st		Specific Action Steps: 43% cited increasing education through providing more classes, training, and resources, 13% increasing the number of healthcare providers and services		There is a lot of engagement and prioritization for Help; however, there is a need for more healthcare providers and services.
Heal: The Taskforce holds many events to support survivors of suicide loss. Survivor support must take into account healing is a period of grief and uncertainty.		Ranking of Heal: Involvement: 3rd Progress: 5th Priority: 4th		Greatest barrier: 3% cited difficulty working with survivors or individuals in a suicidal crisis		Interviewees talked about many events and resources for survivors; however, progress in Heal was ranked last.
Research and Evaluation: The Taskforce understands the importance of research and evaluation. There are opportunities to have ongoing research studies and evaluations.		Ranking of Research and Evaluation: Involvement: 5th Progress: 4th Priority: 5th		Specific Action Steps: 13% cited more research and evaluation		Key informant interviews recognized the importance of research and evaluation, but this area ranked low in involvement, progress, and priority in the survey.
Policy and Advocacy: The Taskforce has challenges with long-term and sustainable funding. While there has been progress in policy initiatives, there is a need for more suicide prevention advocacy.		Ranking of Policy and Advocacy: Involvement: 4th Progress: 3rd Priority: 4th		Greatest barrier: 10% cited policy and organizational challenges Specific Action Steps: 13% cited increasing advocacy efforts to change policy and gain more funding		There is low engagement and priority, but the Taskforce recognizes the need for policy and advocacy to gain funding.
Evaluation Question 3: What is the level of collaboration and communication in the Taskforce?						
Suicide prevention efforts are centered on building and sustaining relationships and partnerships with the community to achieve goals.		Level of communication: Good to excellent (82%). Level of collaboration: High (41%), moderate (24%), low (21%), and none (6%)		No comments related to this theme.		There is good communication but there is room for improvement in collaboration for an even larger impact.

## Data Availability

To protect the privacy of the evaluation participants, interview transcriptions and responses to the survey have been deleted.

## Supplementary Materials

The following supporting information can be downloaded at: https://www.mdpi.com/article/10.3390/ijerph21050565/s1, Table S1: Taskforce’s Five Strategies Handout; Figure S1: Survey.
