# Supplementary material for: A Mixed-Methods Evaluation to Inform the Hawaiʻi Suicide Prevention Strategic Plan
Source: Int J Environ Res Public Health. 2024 Apr 29;21(5):565. doi: 10.3390/ijerph21050565 (PMC11120662; doi:10.3390/ijerph21050565)
Supplement: Supplementary file 1 [file ijerph-21-00565-s001.zip › Supplementary Figure S1_Survey.pdf]

Supplementary Figure S1: Survey

The Strategic Plan has 5 strategy areas, aiming to reduce suicide in Hawai‘i 25% by the year 2025.

1. **Hope -** Increase community awareness around suicide prevention and increase statewide capacity for training.
2. **Help -** Promote suicide prevention as a core component of Hawaii's overall system of care (e.g. intervention, postvention, etc.).
3. **Heal -** Provide survivor support and survivor outreach for those personally touched by suicide, and among those with lived experience.
4. **Research and Evaluation -** Conduct research and evaluation to inform suicide prevention programs, interventions, policies, and overall statewide direction.
5. **Policy and Advocacy -** Ensure policies and protocols set the proper foundation for suicide prevention initiatives.

1) Over the past five years, how much progress have you seen in each of the 5 Strategies?

	A lot of progress	Some progress	A little bit of progress	No progress at all
Hope				
Help				
Heal				
Research & Evaluation				
Policy & Advocacy				

2) Looking forward to the next 3-5 years, please rank the Strategies in order of importance, based on need.

	1st priority	2nd priority	3rd priority	4th priority	5th priority
Hope					
Help					
Heal					
Research & Evaluation					
Policy & Advocacy					

3) For the Strategy you ranked as first priority, please suggest a specific activity and/or action step to make progress on this specific Strategy.

4) Please reflect on your personal involvement with each Strategy:

	Already very involved	Assist/support others	Not involved, but would like to be	Not involved, and not my area of focus
Hope				
Help				
Heal				
Research & Evaluation				
Policy & Advocacy				

5) For the Strategy(ies) you are involved with, what is your greatest success in making progress towards the Strategic Plan goals?

6) For the Strategy(ies) you are involved with, what is your greatest barrier in making progress towards the Strategic Plan goals?

7) How would you characterize the overall level of communication within the Taskforce?

- Excellent
- Good
- Fair
- Poor
- Extremely Poor

8) How would you characterize the overall level of collaboration within the Taskforce?

- Collaboration (members belong to one system; consensus is reached on all decisions)
- Coalition (share ideas; all members have a vote in decision-making)
- Coordination (share information and resources; some shared decision-making)
- Cooperation (provide information to each other; decisions are made independently)
- Networking (aware of each other's activities; decisions are made independently)
- None (no awareness or interaction)

9) Which organization/group do you represent when you participate in the PSHTF? Please check ALL that apply.

- Survivor (i.e., lost a loved one to suicide, survived a suicide attempt)
- Mental health consumer
- Community/non-profit organization
- Faith--based organization
- State agency (e.g., DOH, DPS, DOE, DHS, DOT, etc.)
- Education -- K--12
- Education -- college/university
- Healthcare organization (e.g., hospital, clinic, private practice, etc.)
- Military/DOD/VA
- Law enforcement
- Business (e.g., private sector)
- Community member

- Other: ______________________

10) Please select your age group.
- ≤ 24
- 25-44
- 45-64
- ≥ 65

11) Please select your gender.
- Male
- Female
- Transgender
- Non-binary/non-conforming
- Other
- Prefer not to respond

12) What county are you from?
- Honolulu County
- Kauaʻi County
- Maui County
- Hawaiʻi County

13) Do you have any final comments?
