# Supplementary material for: A Mixed-Methods Evaluation to Inform the Hawaiʻi Suicide Prevention Strategic Plan
Source: Int J Environ Res Public Health. 2024 Apr 29;21(5):565. doi: 10.3390/ijerph21050565 (PMC11120662; doi:10.3390/ijerph21050565)
Supplement: Supplementary file 1 [file ijerph-21-00565-s001.zip › Supplementary Table S1_Handout on Five Strategies .pdf]

Supplementary Table S1: Taskforce's Five Strategies

Goal	Theme	Formal Objective
**Strategy #1 - HOPE**		
**Goal #1A – Community Awareness** *Increase community awareness and communication around suicide prevention as a public health problem that is preventable.*	Communication Strategies	1.1 – Develop a sustained media and communication strategy, incorporating safe messaging guidelines, to raise awareness about suicide prevention.
	Messages	1.2 – Apply messaging and branding techniques to develop prevention messages that incorporate safe messaging that resonate with Hawaiʻi's communities.
	Media Partners	1.3 – Foster ongoing partnerships with media professionals/outlets.
	Internet and social media	1.4 – Expand the use of internet and social media, with safe messaging, to promote suicide prevention.
	Addressing Stigma	1.5 – Increase knowledge of discrimination and stigma (in the context of mental health and suicide prevention), with marginalized/vulnerable communities, including LGBTQ, those with mental illness, and others.
**Goal #1B – Training** *Increase statewide capacity for training across multiple levels and disciplines, including a focus on cultural humility with diverse populations.*	Implementation trainings	1.6 – Increase the number of persons in organizations such as mental health, substance use, education, foster care, juvenile justice programs, health care providers, hospitals, law enforcement, faith-based, community, and workplaces trained to identify and refer people at risk for suicide.
	Resources for training	1.6 – Develop resources to increase the number of suicide prevention trainings available, and to expand the menu of evidence-based trainings.
	Dissemination of trainings	1.7 – Promote trainings for individuals and groups statewide.
**Strategy #2 - HELP**		
**Goal #2** *Promote suicide prevention as a core component of Hawai'i's overall system of care.*	Awareness and training	2.1 – Promote help-seeking across multiple sectors and settings by partnering with State-/county-wide service providers, with emphasis on those that serve individuals at higher risk of suicide.
	Systems Improvement	2.2 – Promote and support adoption and implementation of the Zero Suicide approach within Hawaiʻi's healthcare systems.
	Services during and immediately after crisis	2.3 – Promote the safety and wellbeing of all persons in suicidal crisis.
	Follow-up and continuity of care	2.4 – Implement systems and protocols to enable follow-up and continuity of care.
	Postvention response	2.5 – Increase capacity of communities and care/support systems to respond effectively to suicide clusters and prevent contagion, within their cultural context, and support implementation with education, training, and consultation.
	Policy and regulatory changes	2.6 – Ensure States and county laws facilitate all of the above objectives, and said laws are implemented appropriately.

**Strategy #3 - HEAL**		
**Goal #3A – Survivor Support** *Increase Hope, Help, Healing, and Wellbeing among those personally touched by suicide and among those with lived experience.*	Support groups	3.1 – Ensure that every Suicide Loss and Lived Experience Survivor has access to a safe, nurturing, and sustainable community infrastructure to share/tell their stories, forgive, heal, survive, and thrive.
	Support group facilitators	3.2 – Ensure that every Suicide Loss and Lived Experience Survivor has access to a support group with skilled, trained, and culturally competent support group facilitators.
	Survivor stories	3.3 – Develop, support, maintain and publicize a statewide community-led Suicide Loss and Lived Experience Survivors' "story-telling bank."
	Venues	3.4 – Conduct, support and publicize activities and events that provide opportunities for assistance, validation, and comfort and for connecting and sharing stories.
**GOAL #3B – Survivor Outreach** *Increase State and Community capacity to effectively and efficiently respond to individuals and communities affected by suicide and those with mental health challenges.*	Outreach program	3.5 – Establish, support, maintain, and publicize a community- based statewide Suicide Outreach Program (SOP) on each island.
	Centralized information	3.6 – Develop, post, and publicize a real-time statewide catalogue of counseling, support groups, community events, and other support services and resources for those affected by suicide. Ensure catalog is available and accessible to service providers, health/medical professionals, and the community.
**Strategy #4 - RESEARCH AND EVALUATION**		
**Goal #4** *Conduct and support high-quality research and evaluation to inform suicide prevention programs, interventions, policies, and overall Statewide direction.*	Data systems and sharing	4.1 - Facilitate the development and integration of suicide prevention data systems
	Evaluation of local programs	4.2 – Evaluate existing Hawaiʻi-based suicide prevention programs, policies, and systems, instituting a "culture of evaluation" to ensure provision of high quality and evidence- based interventions.
	Quality Improvement	4.3 – Support service organizations to incorporate organizational evaluation and quality improvement processes.
	Culture and diversity	4.4 – Support high-quality research and evaluation endeavors which will contribute to our understanding of the role of culture in suicide and suicide prevention.
	Pursue new opportunities	4.5 – Pursue funding and partnerships to expand research opportunities, both local and national.
**Strategy #5 - POLICY AND ADVOCACY**		
**Goal #5** *Ensure policies and protocols set the proper foundation for suicide prevention initiatives.*	General Funding	5.1 – Ensure adequate levels of funding and resources for all aspects of the Strategic Plan.
	Advocacy skills and capacity	5.2 – Encourage community members and professionals to engage in advocacy for suicide prevention.
	Organizational protocols and policies	5.3 – Encourage and support agencies and organizations to adopt protocols for suicide prevention, crisis response and management, and postvention.
	Codify the Taskforce	5.4 – Institutionalize suicide prevention networks and collaborations, including the Taskforce.
